# Efficient Near Infrared Light Triggered Nitric Oxide Release Nanocomposites for Sensitizing Mild Photothermal Therapy

**DOI:** 10.1002/advs.201801122

**Published:** 2018-12-11

**Authors:** Xiao Zhang, Jiangfeng Du, Zhao Guo, Jie Yu, Qin Gao, Wenyan Yin, Shuang Zhu, Zhanjun Gu, Yuliang Zhao

**Affiliations:** ^1^ CAS Key Laboratory for Biomedical Effects of Nanomaterials and Nanosafety Institute of High Energy Physics and National Center for Nanosciences and Technology Chinese Academy of Sciences Beijing 100049 China; ^2^ Department of Medical Imaging Shanxi Medical University Taiyuan Shanxi 030001 China; ^3^ College of Materials Science and Optoelectronic Technology University of Chinese Academy of Sciences Beijing 100049 China; ^4^ Engineering Research Center of Molecular and Neuro Imaging of Ministry of Education School of Life Science and Technology Xidian University Xi'an Shaanxi 710126 China

**Keywords:** autophagy, mild photothermal therapy, NIR triggered, nitric oxide release, NO‐sensitized synergistic therapy

## Abstract

Mild photothermal therapy (PTT), as a new anticancer therapeutic strategy, faces big challenges of limited therapeutic accuracy and side‐effects due to uneven heat distribution. Here, near infrared triggered nitric oxide (NO) release nanocomposites based on bismuth sulfide (Bi_2_S_3_) nanoparticles and bis‐*N*‐nitroso compounds (BNN) are constructed for NO‐enhanced mild photothermal therapy. Upon 808 nm irradiation, the high photothermal conversion efficiency and on‐demand NO release are realized simultaneously. Due to the unique properties of NO, enhanced antitumor efficacy of mild PTT based on BNN‐Bi_2_S_3_ nanocomposites is achieved in vitro and in vivo. Mechanism studies reveal that the exogenous NO from BNN‐Bi_2_S_3_ could not only impair the autophagic self‐repairing ability of tumor cells in situ, but also diffuse to the surrounding cells to enhance the therapeutic effect. This work points out a strategy to overcome the difficulties in mild PTT, and has potentials for further exploitation of NO‐sensitized synergistic cancer therapy.

Photothermal therapy (PTT), which converts light energy into heat through light‐absorbing molecules or nanoparticles, has drawn considerable attention in recent years.^1^ To achieve thorough ablation of tumor, the high‐power laser is usually required to heat the tumor over 50 °C, but the painful therapeutic procedures and serious healthy tissue damage limit its practical applications.^2^ Alternatively, mild PTT, which controls the temperature at relatively low level (e.g., 45 °C) in tumor area, has emerged as a new candidate instead.^3^ Unfortunately, the efficacy of mild PTT as a stand‐alone modality remains challenging due to the following reasons: i) heterogeneous distribution of heat in tumor; ii) cellular self‐repairing mechanisms after insufficient heat damage. The utilization of nanoparticles‐mediated mild PTT can partly solve these problems because the high local temperature on the surface of nanoparticles could improve the therapeutic effect.^4^ However, it is still very difficult for nanoparticles to disperse uniformly in the whole tumor so that it may not be realistic to deliver sufficient heat dosage to every tumor cell.^5^ Therefore, it would make a number of cells survive with the help of cellular self‐repairing mechanisms, and thus lead to inexhaustive therapeutic effect and even tumor recurrence and metastasis.^6^

To improve therapeutic efficacy, one possible approach is to combine mild PTT with other treatment modalities.^7^ It was reported that co‐administration of mild PTT and chemo‐drug could significantly enhance the antitumor effect.^8^ While mild PTT improve diffusion and accumulation of drugs in tumor region, the small molecular chemo‐drugs would compensate the insufficient heat damage.^9^ As is known to us, some gaseous molecules, such as nitric oxide (NO), carbon monoxide (CO), and hydrogen sulfide (H_2_S), have good diffusion ability in tumor tissue due to their unique chemical properties.^10^ Meanwhile, these gaseous molecules have been reported to serve as secondary messengers and could regulate many physiological processes.^11^ For instance, NO could directly cause cell death in the relatively high concentration, and sensitize chemotherapy or radiotherapy in the manner of signal pathway regulation.^12^ Some researchers also reported that NO could inhibit the subcellular degraded processes, such as autophagy, to accelerate the apoptotic death of cells.^13^ Therefore, encouraged by its various function in cancer therapy, we envisage that the use of NO in mild PTT could help to circumvent the heterogeneous distribution problem and enhance therapeutic efficacy.

Since NO participates in various kinds of physiological activities, construction of the targeted delivery system instead of the existing sustained‐release NO donor is extremely valuable.^14^ Recently, near infrared light triggered release system based on two‐photon absorption nanoparticles or upconversion nanoparticles became a competitive candidate due to the ideal penetration depth of NIR light.^15^ However, these nanosystems suffered from low quantum yield, resulting in low NO releasing efficiency. On the contrary, the photothermal conversion efficiency of PTT agents could reach nearly 30% in NIR region, which provided enough energy for NO release through physical or chemical reaction.^16^ Due to this advantage, some groups have investigated photothermal conversion based NO release systems as well as the combined therapeutic effects of NO with PTT.^17^ However, most of them were not real on‐demand release systems because the NO donors were sustained‐release type. During circulation, these NO delivery systems would release NO spontaneously and cause the unexpected side‐effects. Another important issue was that the previous reported photothermal responsive NO platforms usually used high temperature to promote NO release as well as achieve efficient thermal ablation of tumor. But as we discussed above, high temperature PTT has limited practicality in clinic. Therefore, how to fabricate the real on‐demand NIR‐responsive NO release system and apply them for mild PTT enhancement are still problems that needs to be solved. These scientific issues are of great importance in both advanced NO donors design and NO‐mediated synergistic cancer therapy.^18^

In this work, we formulated the efficient NIR‐triggered NO release nanocomposites based on bismuth sulfide (Bi_2_S_3_) nanoparticles and hydrophobic NO donor, *N*,*N*′‐Di‐sec‐butyl‐*N*,*N*′‐dinitroso‐1,4‐phenylenediamine (BNN), which was loaded on the surface of Bi_2_S_3_ nanoparticles (**Scheme** **1**). Upon NIR laser irradiation, Bi_2_S_3_ nanoparticles could act as the heating source in cells, resulting in significant rise of the local temperature in cancer cells while keeping the apparent temperature at low level in surroundings to realize mild PTT. This feature also makes it ideal for NIR‐triggered NO release. Herein, we employ BNN as the NO donor, which can avoid the unexpected NO release in physiological condition because BNN is relatively stable below 60 °C. Upon NIR irradiation, the high local temperature on the surface of Bi_2_S_3_ nanoparticles can be achieved, leading to the decomposition of BNN as well as NO release. These properties enable the combination of BNN and Bi_2_S_3_ to serve as both mild PTT agents and on‐demand NO donors. Based on the novel nanocomposites, we for the first time realized NO‐sensitized mild PTT in vitro and in vivo, and further illustrated their synergistic mechanisms. The as‐prepared BNN‐Bi_2_S_3_ nanocomposites offer a new strategy for the design of NO delivery system based on nanotechnology, and it also has potentials for further exploitation as a powerful platform for the NO and PTT synergistic antitumor therapy.

**Scheme 1:**
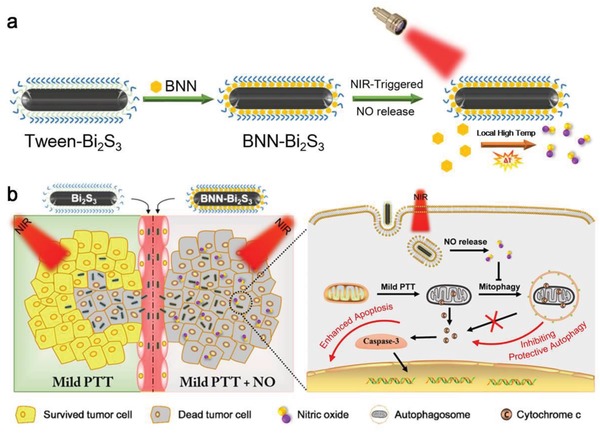
a) Schematic illustration of synthetic procedure and NIR‐triggered NO release property of BNN‐Bi_2_S_3_. b) Synergistic mechanism of NO and mild PTT in cancer therapy.

The structure of BNN‐Bi_2_S_3_ is illustrated in **Figure** **1**a. The core Bi_2_S_3_ nanorods were synthesized according to the modified thermal decomposition method.^19^ The transmission electron microscope (TEM) and scanning electron microscope (SEM) images of the as‐prepared Bi_2_S_3_ nanorods exhibited a uniform size with length of ≈80 nm and diameter of about 8 nm (Figure 1b and Figure S1a, Supporting Information). The X‐ray diffraction (XRD) and energy dispersive spectroscopy (EDS) were conducted to confirm the purity and chemical compositions (Figures S1b and S2, Supporting Information). To get well dispersibility in physiological solution, a commercial surfactant Tween‐20 was modified on the surface of Bi_2_S_3_ nanorods through hydrophobic interaction. As an amphiphilic molecule, the outside hydrophilic chain of Tween‐20 ensured good dispersibility and the inside hydrophobic chain provided the storage space for organic molecules. Afterward, the water‐insoluble NO donor BNN was synthesized following the work of Chen and co‐workers.^20^ ^1^H NMR spectrum was carried out to confirm the successful synthesis of BNN (Figure S3, Supporting Information). Then, BNN was loaded through hydrophobic–hydrophobic interaction. The UV–vis absorption spectra, Fourier transform infrared (FT‐IR) spectra, and thermal gravity analysis (TGA) verified the successful loading of BNN on the surface of Bi_2_S_3_ nanorods (Figure 1c and Figure S4, Supporting Information). The saturation adsorption of BNN could reach nearly 50 wt% (Figure S4a, Supporting Information). After BNN loading, the hydrodynamic size of BNN‐Bi_2_S_3_ nanocomposites was slightly larger than that of Bi_2_S_3_ nanorods, and the zeta potential of BNN‐Bi_2_S_3_ was about −16 mV (Figure 1d). In addition, the BNN‐Bi_2_S_3_ nanocomposites could be well stored in PBS solution without obvious BNN leakage or significant aggregation (Figures S4d and S5, Supporting Information). The above data suggested that BNN‐Bi_2_S_3_ nanocomposites were suitable candidates for biological applications.

**Figure 1:**
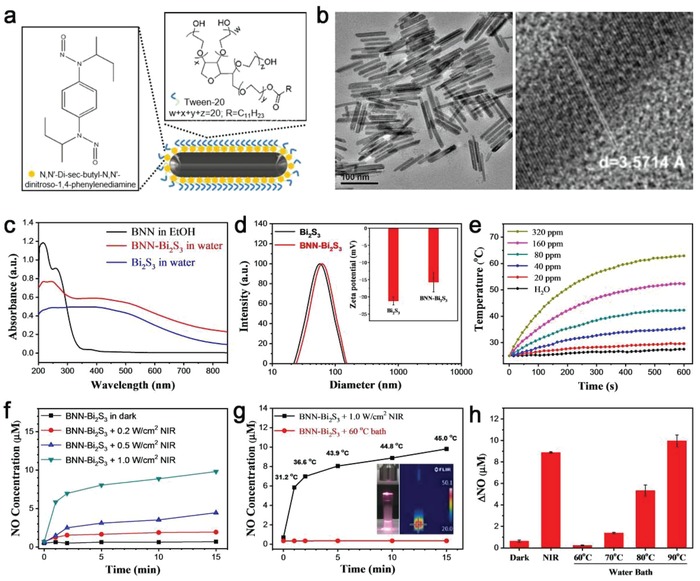
Characterization and performance monitoring of BNN‐Bi_2_S_3_ nanocomposites. a) Structural illustration of BNN‐Bi_2_S_3_ nanocomposites. b) TEM image of Bi_2_S_3_ nanorods (left); high resolution TEM image of a single Bi_2_S_3_ nanorod (right). c) UV–vis absorption spectra of Bi_2_S_3_, BNN, and BNN‐Bi_2_S_3_. d) Hydrodynamic diameters of Bi_2_S_3_ and BNN‐Bi_2_S_3_ measured by dynamic light scattering. Inset: zeta potential of Bi_2_S_3_ and BNN‐Bi_2_S_3_. e) The photothermal profiles of pure water and aqueous dispersions of BNN‐Bi_2_S_3_ with different concentrations under 808 nm laser irradiation with a power density of 1.0 W cm^−2^. f) Generation of NO from BNN‐Bi_2_S_3_ under 808 nm laser irradiation with different power density. g) The amount of released NO from BNN‐Bi_2_S_3_ under 808 nm laser irradiation or in water bath at 60 °C. The temperature was monitored during the process. Inset: the photograph and infrared thermal image of BNN‐Bi_2_S_3_ aqueous dispersion under NIR irradiation. h) The NO concentration in supernatant in different groups. The data points are shown as mean value and standard deviation, *n* = 3.

Due to its broad absorption in NIR region, the NIR‐triggered NO release property of BNN‐Bi_2_S_3_ was investigated. First of all, the temperature of BNN‐Bi_2_S_3_ aqueous solution was recorded upon NIR irradiation while PBS was set as control (Figure 1e). The photothermal conversion efficiency of BNN‐Bi_2_S_3_ nanocomposites was calculated to be 33.7% according to the obtained data (Figure S6, Supporting Information). This high photothermal conversion efficiency was the foundation to provide enough energy for the following decomposition of NO donors. As shown in Figure 1f, the concentration of released NO from BNN‐Bi_2_S_3_ under NIR irradiation (1.0 W cm^−2^) could reach nearly 10 × 10^−6^ m after 15 min. Compared to the other NIR‐triggered NO releasing nanomaterials, this NO delivery and release system exhibited better NO release efficiency (Table S1, Supporting Information). As mentioned earlier, the reasonable surface modification by the amphiphilic Tween‐20 provided enough storage space for the organic NO donors, ensuring the efficient NO delivery. More importantly, the high photothermal conversion efficiency of Bi_2_S_3_ nanocomposites provided enough energy for BNN decomposition. These two aspects are the key characters in the BNN‐Bi_2_S_3_ nanocomposites as well as in other controllable NO delivery materials.

Next, we monitored the temperature throughout the NO release process using an infrared thermal imager. The temperature of the samples kept no more than 46 °C under NIR irradiation at 1.0 W cm^−2^ (Figure 1g). However, direct heating (water bath) the samples at 60 °C did not lead to remarkable NO release. Further increase of heating temperature to 90 °C resulted in gradually raising NO release (Figure 1h). This contradictory phenomenon could derive an interesting corollary that the Bi_2_S_3_‐based photothermal conversion was totally different from the usual water bath heating process. As reported before, the local temperature on the surface of light absorptive nanoparticles was much higher than the surrounding temperature.^21^ In this case, the high local temperature on the surface of BNN‐Bi_2_S_3_ resulted in BNN decomposition and release of NO while the apparent temperature in surroundings was kept at 46 °C. This high local temperature induced pyrolysis reaction of BNN that happened on the surface of nanoparticles have two advantages: i) NO could release efficiently under the mild condition with minimal heat damage to normal tissues; ii) only local heat could cause NO release, which avoided the undesired NO release to minimize the side‐effects of NO. The stability of the as‐prepared BNN‐Bi_2_S_3_ nanocomposites was tested in physiological condition. The result showed little NO release after stirring for 24 h (Figure S7, Supporting Information). Therefore, utilizing this high local temperature strategy could help to develop a real on‐demand NO release system, and further potentially promote the development of the general drug delivery system in biomedical field.

The biocompatibility and membrane‐permeability are both important for NO delivery system. At the beginning, the cytotoxicity of Bi_2_S_3_ and BNN‐Bi_2_S_3_ were evaluated on the three kinds of human cells. The results showed that neither Bi_2_S_3_ nor BNN‐Bi_2_S_3_ has obvious cytotoxicity (Figure S8, Supporting Information). Furthermore, intracellular distribution of BNN‐Bi_2_S_3_ was visualized through dark‐field imaging, and then quantified through inductively coupled plasma mass spectrometry (Figure S9, Supporting Information). Herein, the intracellular NO release property of BNN‐Bi_2_S_3_ was evaluated using fluorescent probe (DAF‐FM DA). Both of the fluorescence images and flow cytometry results demonstrated that only the cells treated with BNN‐Bi_2_S_3_ and exposed to NIR irradiation showed obvious green fluorescence in the cytoplasm, successfully confirming the efficient generation of NO in cells (Figure S10, Supporting Information). More importantly, the NO signal could be observed not only in the irradiated cells but also in the unirradiated cells out of laser spot, indicating the high diffusivity of NO among cells (Figure S11, Supporting Information).

To further investigate the diffusion capacity of NO in vitro, a multicellular tumor spheroid model (MCTS) based on BEL‐7402 cells was established in this work. As shown in **Figure** **2**a, BEL‐7402 cells were cultured in agarose coated 96‐well plate to form tumor spheroid, and then co‐incubated with BNN‐Bi_2_S_3_ nanoparticles for 24 h. After incubating, the frozen sections of the MCTS were prepared. The distribution of BNN‐Bi_2_S_3_ nanoparticles was observed using dark‐field microscope. It is obvious that the signal of BNN‐Bi_2_S_3_ nanoparticles could either appear on the periphery of the MCTS or the interior surface of some porous channels (Figure 2b). This result verified the restricted intercellular diffusion of nanoparticles in tumor spheroid, which is a big challenge of nanomaterials for solid tumor treatment according to the previous reports.[[qv: 6d,22]] Although nanoparticles could hardly distribute homogeneously, we supposed that the gas molecules from carriers could diffuse freely throughout the whole tumor because of its well diffusion ability across cell membrane. As shown in Figure 2c, MCTS was imaged from the bottom to top by *Z*‐axis step scanning using confocal microscope. Interestingly, the signal of NO probe in the group treated with both BNN‐Bi_2_S_3_ and NIR could be observed throughout the whole tumor spheroid. The flow cytometry results further confirmed that NO diffused to most of the tumor cells in the MCTS (Figure 2d). As mentioned earlier, BNN‐Bi_2_S_3_ nanoparticles were located only on the surface of the MCTS, which had limited penetration ability to the core region of the spheroid. However, as a gaseous molecule, NO exhibited good tumor penetration ability. The utilization of NO could avoid the limitation of nanoparticles in medical applications, including the poor penetration capacity and the inhomogeneous distribution in solid tumor.

**Figure 2:**
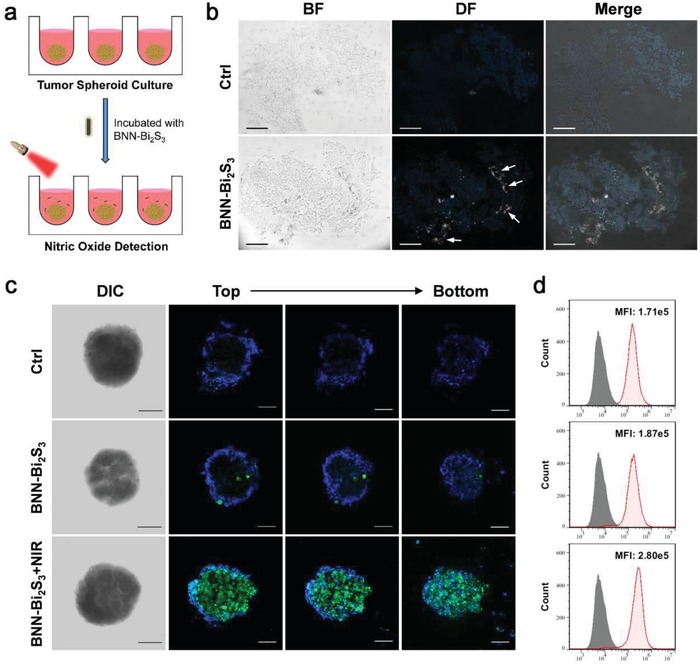
Evaluation of NO diffusing capacity in MCTS model. a) Schematic illustration of NIR‐stimuli NO release from BNN‐Bi_2_S_3_ in 3D tumor spheroid model. b) The bright field and dark field images of the frozen sections of tumor spheroids with or without BNN‐Bi_2_S_3_ treatment. Arrows represent dark‐field signal of BNN‐Bi_2_S_3_ nanoparticles. The scale bars are 100 µm. c) Confocal images of tumor spheroid with different treatments. The cells were stained with Hoechst 33342 (blue) and DAF‐FM (green). The concentration of BNN‐Bi_2_S_3_ nanoparticles is 20 µg mL^−1^. The irradiation power is 0.5 W cm^−2^, for 10 min. d) Flow cytometry analysis of BEL‐7402 cells trypsinized from the tumor spheroids in different groups. The shadow curve is the unstained negative control.

Then, the therapeutic potentials of BNN‐Bi_2_S_3_ nanocomposites were examined on BEL‐7402 cell lines in vitro. The cell viability decreased obviously with the increase of the concentration and NIR power density (**Figure** **3**a). Moreover, the inhibition ability of BNN‐Bi_2_S_3_ was much higher than that of Bi_2_S_3_ when their temperatures were kept the same (Figure 3b). The results clearly showed that mild PTT, to some extent, could inhibit tumor cells while the released NO from BNN‐Bi_2_S_3_ could intensify the PTT therapeutic effect. The apoptosis/necrosis staining was further conducted to evaluate the cell killing efficacy. As shown in Figure 3c, no obvious early apoptosis or late apoptosis cells were detected in the groups treated by PBS, Bi_2_S_3,_ or BNN‐Bi_2_S_3_ alone. On the contrary, when the cells were treated with Bi_2_S_3_ under NIR irradiation, the early apoptosis cells and late apoptosis cells increased to 14.7% and 12.4%, respectively. The apoptosis/necrosis promotion was more obvious in the group of cells treated with BNN‐Bi_2_S_3_ under NIR irradiation, which was calculated to be 23.8% and 24.1%. (Figure 3d). These results revealed that NO released from BNN‐Bi_2_S_3_ under NIR irradiation could enhance the PTT effect by means of promoting cell apoptosis.

**Figure 3:**
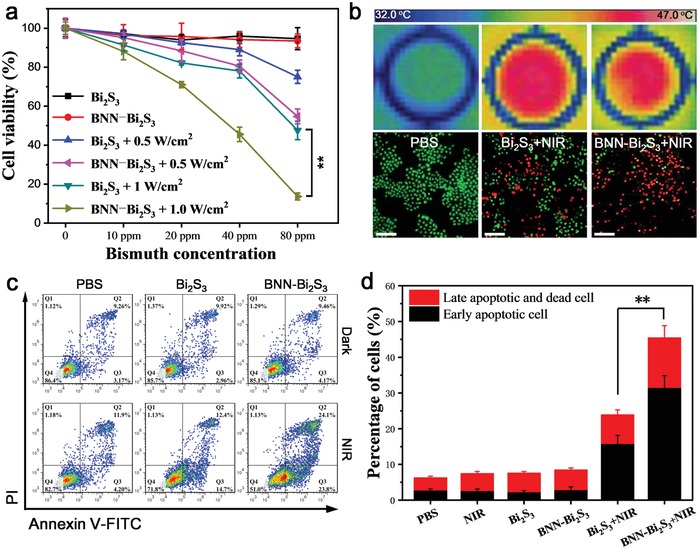
Synergistic effect of mild PTT and NO based on BNN‐Bi_2_S_3_ nanocomposites. a) Cell viability of BEL‐7402 cells treated by Bi_2_S_3_ or BNN‐Bi_2_S_3_ with different concentrations under 808 nm laser irradiation or in dark condition. b) Thermal images and the live–dead stained images of BEL‐7402 cells with different treatment. The scale bars are 100 µm. c) Flow cytometry was examined to determine the percentages of apoptosis and necrosis cells on BEL‐7402 cells with different treatment. d) Statistical data of percentage of apoptosis and late apoptosis/necrosis cells under different treatments. The data are shown as mean value and standard deviation, *n* = 3. The concentration of Bi_2_S_3_ or BNN‐Bi_2_S_3_ keeps 80 ppm (Bi^3+^). The laser power density is 1.0 W cm^−2^ (10 min). ^**^ *p* < 0.01.

Next, the molecular mechanism was investigated. It is generally acknowledged that the in‐depth mechanisms of NO‐associated synergistic therapy should be investigated by using molecular and genetic methodologies.^18^ In addition to the toxic effect, the biological regulation functions of NO in tumor therapy have also attracted much attention, such as improving hypoxia and inhibiting autophagy. According to the recent research which suggested the occurrence of autophagy in hyperthermia‐induced apoptosis,^23^ we detected the autophagy levels of cancer cells after different treatment using Cyto‐ID Autophagy Detection Kit. As shown in **Figure** **4**a, green puncta (represented autophagosome) remarkably accumulated in the cells after being treated with Bi_2_S_3_ under NIR irradiation compared to the other groups. The statistical data from flow cytometry verified the increased autophagy level after mild PTT (Figure S12, Supporting Information). Besides, Acridine Orange (AO) was also used for autophagy detection, which showed a characteristic increase in red fluorescence that indicated the appearance of acidified autophagosome (Figure 4b and Figure S13, Supporting Information). Results from Western Blot confirmed that mild PTT treatment increased endogenous LC‐3II levels significantly while BNN‐Bi_2_S_3_+NIR group exhibited slight accumulation of LC3‐II (Figure 4c). The conversion of LC3‐I to LC3‐II is widely used to indicate the activation of autophagy. Because the steady state levels of LC3‐II are affected by both autophagsome formation and degradation, the expression level of degradation‐associated proteins should also be evaluated. The results showed that p62 levels was downregulated remarkably after mild PTT, but decreased slightly in BNN‐Bi_2_S_3_+NIR group. It was obvious that mild PTT indeed induced autophagy in cytoplasm and NO arrested PTT‐induced autophagy. As a consequence, we speculated that the activation/inhibition of autophagy played an important role in cell death during PTT. To reveal the function of autophagy in PTT‐induced apoptosis process, we used autophagy inducer rapamycin (Rapa) or inhibitor 3‐methyladenine (3‐MA) during PTT and made a comparison with Bi_2_S_3_ or BNN‐Bi_2_S_3_ under NIR irradiation. The cell viability results demonstrated that Rapa could reduce the therapeutic effect of PTT. On the contrary, when autophagy was inhibited by 3‐MA, the cell viability decreased to the same level as the cells treated with BNN‐Bi_2_S_3_ under NIR irradiation (Figure S14, Supporting Information). Taken together, we could draw a primary conclusion that autophagy might protect the cell death in mild PTT, while NO functioned to prevent this protective effect and aggravate the heat‐induced injury.

**Figure 4:**
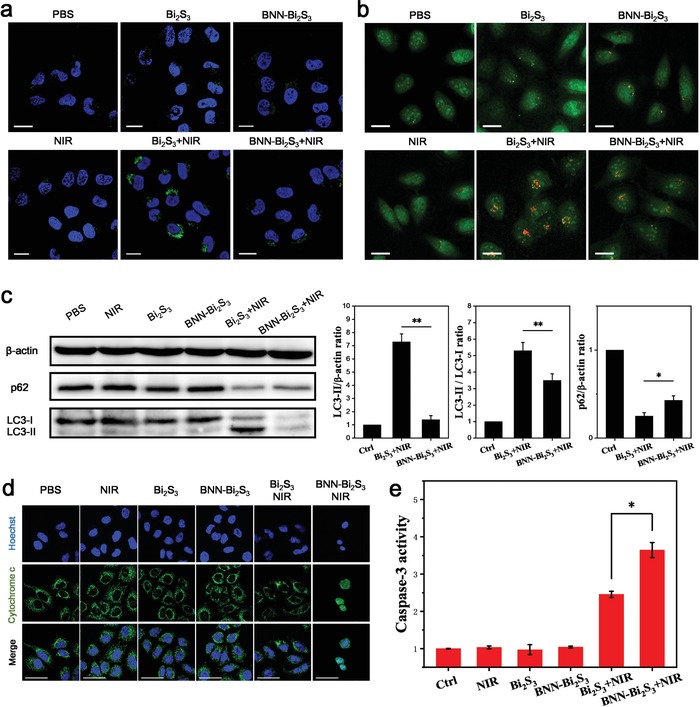
NO inhibits protective autophagy to sensitize photothermal therapy. a) Confocal microscopy images of BEL‐7402 cells with different treatment. The cells were stained with Hoechst 33342 (blue) and CYTO‐ID Autophagy detection kit (green). b) Confocal microscopy images of BEL‐7402 cells stained with acridine orange (AO). All the scale bars are 20 µm. c) Western blot and its analysis of LC3‐II, LC3‐I, and p62 of BEL‐7402 cells with different treatment. d) Immunofluorescent staining with cytochrome c of BEL‐7402 cells in each group (green). The nuclei were labeled with Hoechst 33342 (blue). The scale bars are 50 µm. e) Relative caspase‐3 activity in BEL‐7402 cells with different treatment. The value of control was set to 1. All the statistical data are shown as mean value and standard deviation, *n* = 3. *p* values in (c,e) were calculated by Student's *t*‐test (^**^ *p* < 0.01 or *p < 0.05).

Motivated by the above results, we thus investigated the autophagy‐based protective mechanism under mild PTT and revealed the function of NO in this mechanism. As reported previously, autophagy could degrade the damaged mitochondria under heat stress, and this mitophagy process would prevent the content of mitochondria from releasing to the cytoplasm.^24^ Herein, cytochrome c, as a promoter in mitochondria damage‐induced apoptosis, was detected as the marker of mitochondria damage and apoptosis activation. As shown in Figure 4d, the uniformly dispersed cytochrome c spots were observed in cells treated with BNN‐Bi_2_S_3_ under NIR irradiation, indicating the complete release of cytochrome c from mitochondria into cytosol. The released cytochrome c would further activate the caspase‐3 mediated apoptosis pathway (Figure 4e). Based on these results, we found that autophagy is a prosurvival pathway in mild PTT which functions to degrade the damaged mitochondria, reduce the release of cytochrome c, and inhibit the activity of caspase‐3. The introduction of NO could downregulate autophagy level to reduce its protective effect. Protective autophagy was generally considered to be a survival mechanism after PTT and other various therapeutic modalities.[[qv: 23b,25]] Therefore, regulating autophagy by the related inducers and inhibitors has become a practical strategy for tumor therapy. Our results proved that NO provided a potential approach to modulate autophagy and enhance the therapeutic efficacy of PTT and other therapies. This would open a new way for the autophagy regulation and also extend the application of NO for cancer treatment.

Having proved the synergistic mechanism of NO and PTT, we further investigated their tumor inhibition performance in vivo. The female BEL‐7402 tumor bearing mice were randomly divided into six groups: i) PBS injection; ii) laser; iii) Bi_2_S_3_ injection; iv) Bi_2_S_3_ + laser; v) BNN‐Bi_2_S_3_ injection; vi) BNN‐Bi_2_S_3_ + laser. The dose of injected Bi_2_S_3_ or BNN‐Bi_2_S_3_ was 20 mg kg^−1^, and the NIR exposed time was 10 min (0.35 W cm^−2^). The temperature of the exposed area was recorded using an infrared thermal imager during the PTT process. The tumor temperature of the groups iv) and vi) increased to 46 °C under NIR irradiation while the tumor temperature of the groups ii) only increased a little (**Figure** **5**a and Figure S15, Supporting Information). After NIR exposure, the concentration of NO in tumor tissue lysate of each group was measured using the typical Griess reagent method. The results exhibited that the concentration of NO in group vi) was obviously higher than that in the other groups, indicating the release of NO from BNN‐Bi_2_S_3_ under NIR irradiation in vivo (Figure 5b). The tumor volumes of each group were recorded every two days after PTT (Figure 5c). Tumors in the PBS injection group grew rapidly while only laser, Bi_2_S_3_ or BNN‐Bi_2_S_3_ had no obvious suppressing influence on tumor volumes and tumor weight compared to the control group (Figure 5d and Figure S16, Supporting Information). In contrast, the group iv) and vi) showed significant tumor inhibition, and the average tumor weight in group iv) and vi) decreased to 47% and 24% compared to the control group. We found that Bi_2_S_3_ based mild PTT could achieve tumor inhibition, but tumor had the trend of recurrence one week after treatment. However, the combination of NO and PTT based on BNN‐Bi_2_S_3_ could obtain completed tumor inhibition, demonstrating that the synergistic effect of NO and mild PTT can eradicate tumor cells effectively. In the antitumor experiment, the therapeutic temperature in tumor area was controlled at about 46 °C to maintain the mild PTT condition. However, the therapeutic effect of PTT alone was unsatisfied due to the reasons including the self‐repairing mechanisms of cells and heterogeneous distribution of PTT heat seeds. The distribution of Bi_2_S_3_ nanoparticles in tumor site was confirmed by both X‐ray computed tomography (CT) and multispectral optoacoustic tomography (MSOT) after intratumor injection (Figure S17, Supporting Information). The heterogeneous distribution of Bi_2_S_3_ nanoparticles would cause heterogeneous distribution of heat in tumor site, which might result in unsatisfied therapeutic effect. On the contrary, although the distribution of BNN‐Bi_2_S_3_ nanocomposites in tumor was still heterogeneous, the well‐diffused NO could distribute uniformly in the whole tumor to synergistically treat tumor through inhibiting the autophagic self‐repairing process. Thus, using BNN‐Bi_2_S_3_ nanocomposites as the PTT agents could remarkably increase the therapeutic effect. The pathological sections of all the tumor tissues were further investigated. Hematoxylin and eosin (H&E) staining and terminal deoxynucleotidyl transferase dUTP nick end labeling (TUNEL) staining showed that mild PTT inhibited tumor growth in comparison with the control group, and the synergy of NO and mild PTT enhanced the inhibition effect (Figure 5e). In addition, the immunofluorescent method was conducted to detect the expression of LC3‐II in tumor tissues. As shown in Figure 5e, a stronger green fluorescence was observed in group iv) compared to the control group, while the tumor tissue of group vi) exhibited week fluorescence. Besides, none of the groups had obvious changes in body weight and other organs, indicating no extreme toxicity with these treatments in vivo (Figures S18 and S19, Supporting Information). These results confirmed that the combination of NO and mild PTT could inhibit PTT‐induced autophagic self‐repairing, enhancing the therapeutic efficacy with no obvious side‐effects in vivo.

**Figure 5:**
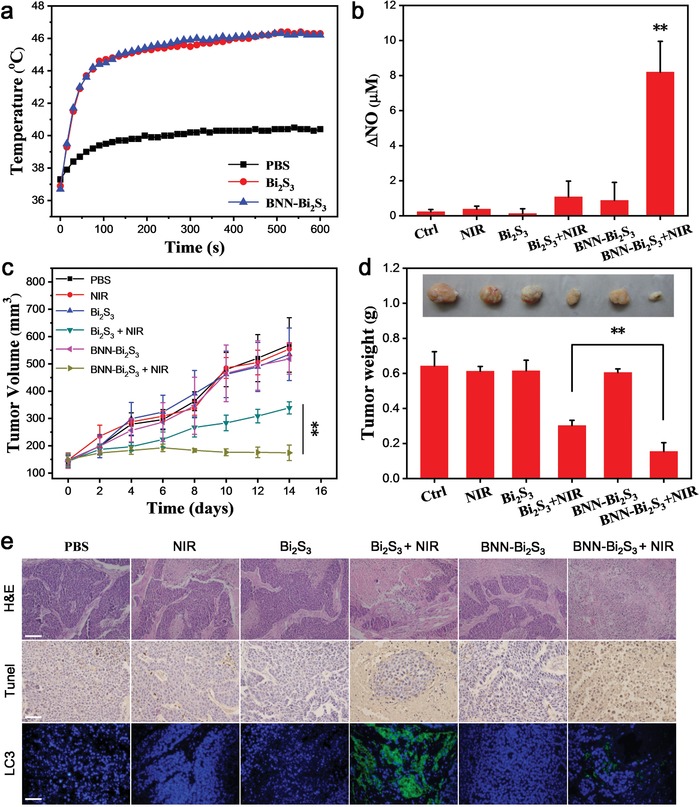
Synergistic antitumor effect of photothermal therapy and NO in vivo. a) The temperature of tumor obtained from thermal infrared imager. b) The changes of NO concentration in each group after treatment. The data are shown as mean value and standard deviation, *n* = 3. c) Tumor volume curves of different groups of mice after various treatments (four mice for each group). d) The average tumor weight in each group. Inset: Photo of the representative tumors collected from different groups at the end of treatment. e) Pathological features of tumor tissues in representative mice. Tumor tissues were H&E stained (top), TUNEL stained (middle), and LC3‐II antibody Stained (bottom); the entire nucleus was stained with Hoechst 33342. All the scale bars are 100 µm. *p* values in (b),(c) were calculated by Student's *t*‐test (^**^ *p* < 0.01).

In conclusion, a new therapeutic strategy based on the well‐designed nanocomposites containing photothermal conversion nanoparticles and NO donors was established. Combined the photothermal conversion effect of Bi_2_S_3_ nanoparticles with the thermally decomposition ability of NO donor, BNN‐Bi_2_S_3_ nanocomposites could act as both photothermal agent for PTT and drug carrier for NIR triggered NO release. The NIR light generated NO could diffuse homogeneously in solid tumor to impair the self‐repairing ability of tumor cells in mild PTT. As a result, BNN‐Bi_2_S_3_ nanocomposites exhibited more effective tumor inhibition ability than Bi_2_S_3_ alone both in vitro and in vivo. In a word, the newly‐prepared nanocomposites could simultaneously realize a controllable NO release and PTT, and the mechanism of their synergistic effects in cancer therapy was also illustrated. Consequently, these features endow BNN‐Bi_2_S_3_ nanocomposites with potentials for further exploitation as a powerful platform for NO‐sensitized synergistic antitumor therapy.

## Conflict of Interest

The authors declare no conflict of interest.

## Supporting information

Supplementary
