# Supplementary material for: Efficient Near Infrared Light Triggered Nitric Oxide Release Nanocomposites for Sensitizing Mild Photothermal Therapy
Source: Adv Sci (Weinh). 2018 Dec 11;6(3):1801122. doi: 10.1002/advs.201801122 (PMC6364593; doi:10.1002/advs.201801122)
Supplement: Supplementary file 1 — Supplementary [file ADVS-6-1801122-s001.pdf]

Copyright WILEY-VCH Verlag GmbH & Co. KGaA, 69469 Weinheim, Germany, 2018.

Supporting Information

for *Adv. Sci.,* DOI: 10.1002/advs.201801122

Efficient Near Infrared Light Triggered Nitric Oxide Release Nanocomposites for Sensitizing Mild Photothermal Therapy

Xiao Zhang, Jiangfeng Du, Zhao Guo, Jie Yu, Qin Gao, Wenyan Yin, Shuang Zhu, Zhanjun Gu, and Yuliang Zhao**

Copyright WILEY-VCH Verlag GmbH & Co. KGaA, 69469 Weinheim, Germany, 2016.

Supporting Information

An Efficient Near Infrared Light Triggered Nitric Oxide Release Nanocomposites for Sensitizing Mild Photothermal Therapy

Xiao Zhang, Jiangfeng Du, Zhao Guo, Jie Yu, Qin Gao, Wenyan Yin, Shuang Zhu, Zhanjun Gu*, and Yuliang Zhao*

Experimental Section

Reagents: All chemicals were purchased from Sigma-Aldrich unless specified otherwise. Calcein-AM (CA), propidium iodide (PI), Annexin V-FITC Apoptosis Detection Kit, and Cell Counting Kit-8 (CCK-8) were provided by Dojindo Laboratories in Japan. DAF-FM and Hoechst 33342 were supplied from Beyotime Biotechnology. All the reagents were used without further purification. DI water was obtained by an 18.2 MΩ recirculating deionized water system (arium pro DI, Sartorius).

Synthesis of Bi_2S_3 nanorods: 2 mmol of $Bi(NO_3)_3$ was dispersed in 10 mL of OA and 10 mL of OM in a 100 mL flask. The solution was heated to 170 ^oC with stirring under Ar_2 atmosphere and kept at this temperature for 40 min. After the solution turned grey, a solution of 10 mmol of sulfur in 5 mL of OM was injected swiftly. The reaction was kept at 170 ^oC for 10 min and then stopped by injecting 40 mL of cold cyclohexane. The mixture was centrifuged at 8 000 rpm for 3 min, and the precipitate was collected and washed with hexane and ethanol (1:1 v/v) for 3 times.

Surface modification of Bi_2S_3 using Tween-20: 100 μL of Tween-20 was dispersed in 5 mL of cyclohexane and ultrasonic oscillated for 10 min. And then, 50 mg of the as-prepared Bi_2S_3 nanorods was dispersed in 15 mL of cyclohexane, ultrasonic oscillated and injected into the previous solution. The mixture was heated to 70 ^oC to evaporate cyclohexane. When the cyclohexane was completely removed and the solution became oily, 40 mL of DI water was added and the mixture solution was ultrasonic oscillated. The mixed solution was sequentially kept at 70 ^oC to evaporate residual cyclohexane. The final product was obtained through centrifugation at 12 000 rpm for 10 min, and then washed with ethanol and water alternately for 3 times.

Synthesis of NO donor: N,N'-Di-sec-butyl-N,N'-dinitroso-1,4-phenylenediamine (BNN) was synthesized as follows. 10 mmol of N,N'-bis-sec-butylamino-p-phenylenediamine (BPA) was diluted into 20 mL of ethanol, and then 20 mL of DI water containing 8.28 g of $NaNO_2$

was added under stirring and Ar_2 protection. After half hour, 20 mL of 6 M HCl was added slowly. Then, the mixture solution became yellow and precipitation appeared. After 6 h, the product was collected by centrifugation and washed with ethanol/water (1:1 v/v) and water in turn for 3 times. The solid product was freezing dried in dark condition.

BNN loading and releasing: BNN was loaded in the Tween-20 layer on the surface of Bi_2S_3 through an impregnation method. 20 mg of Tween-20 coated Bi_2S_3 was dispersed in 20 mL of DI water, and then a solution of 20 mL of ethanol containing different amount of BNN was added. After stirring for 12 h in dark condition, the product was separated through centrifugation, and further washed by ethanol and water for several times to remove excess BNN. Meanwhile, the UV-Vis absorption spectra of the BNN supernatant collected after the first centrifugation procedure was measured. The loading percentage was calculated according to the standard curve of BNN in water/ethanol (1:1) solution. To test the stability, we investigated the release behavior of BNN from BNN-Bi_2S_3 in PBS solution at 37 ^{o}C. Briefly, 10 mg of BNN-Bi_2S_3 was dispersed in 20 mL of PBS solution containing 33 % ethanol, and then stirred at 37 ^{o}C. After stirring for the specific time (1h, 2h, 3h, 6h, 12h and 24h), 200 μL of the solution was taken out. The samples were centrifugation at 12 000 rpm for 5 min, and the supernatant was collected to detect the concentration of BNN by UV-Vis absorption spectra. The final concentration was calculated according to the standard curve of BNN in PBS solusion (33% ethanol).

Calculation of the photothermal conversion efficiency: The energy conversion for the system can be expressed by equation 1

$$\sum_i m_i c_{p,i} \frac{dT}{dt} = Q_{NP} + Q_B - Q_{sur} \quad (1)$$

where m and C_p are the mass and heat capacity, respectively. T refers to the solution temperature. Q_{NP} represents the energy of nanoparticles, Q_B is the baseline energy of the

sample container, and Q_{sur} is the heat conduction from the surface to air. Q_{NP} could be calculated by equation 2.

$$Q_{NP} = I(1-10^{-A_{808}})\eta \tag{2}$$

where I is the laser power, η is the photothermal conversion efficiency, and A_{808} is the absorbance of BNN-Bi_2S_3 at wavelength of 808 nm. Q_{sur} is given by equation 3.

$$Q_{sur} = hS(T - T_r) \tag{3}$$

where h is heat transfer coefficient, S means the surface area of the sample container, and T_r is the room temperature during the experiment. The heat input and output becomes balance when the system temperature reach a maximum. So, the photothermal conversion efficiency η can be calculated by the following equation 4.

$$\eta = \frac{hS(T_{max} - T_r) - Q_B}{I(1-10^{-A_{808}})} \tag{4}$$

where Q_B was calculated to be 58.2 mW in an independent experiment using the quartz cuvette filling with water. The (T_{max}-T_r) was 20.3 ^{o}C according to Figure S6 in supporting information. I is 1.0 W cm^{-2}, A_{808} was 0.358 from UV-VIS spectrophotometer. To calculate hS, θ is defined as the equation below.

$$\theta = \frac{T - T_r}{T_{max} - T_r} \tag{5}$$

a sample system time constant $\boldsymbol{\tau_s}$ is also introduced

$$\tau_s = \frac{\sum_i m_i c_{p,i}}{hS} \tag{6}$$

Substitute equation 3 and 6 to the differential of equation 5

$$\frac{d\theta}{dt} = \frac{1}{\tau_s}\left[\frac{Q_{NP} + Q_B}{hS(T_{max} - T_r)} - \theta\right] \tag{7}$$

In cooling process, the laser is off, so Q_{NP} + Q_B = 0, yield

$$\mathrm{d}t = -\tau_s \frac{\mathrm{d}\theta}{\theta} \tag{8}$$

Integral the equation 8, giving the expression

$$t = -\tau_s \ln\theta \tag{9}$$

So, time constant τ_s is determined to be 344.4 as the slope from the cooling period versus negative natural logarithm of θ (Supplementary Fig. S6b). Additionally, m is 1.0 g and C is 4.2 J g^{-1}. Thus, the photothermal conversion efficiency of BNN-Bi_2S_3 can be calculated to be 33.8 %.

NO detection of BNN-Bi_2S_3 under NIR irradiation: A fluorescent probe 2,3-Diaminonaphthalene (DAN, Sigma) was used to detect the release of NO from BNN-Bi_2S_3. The standard curve should be established using $NaNO_2$ before the measurement. The as-prepared BNN-Bi_2S_3 was dispersed in DI water (100 ppm, Bi concentration) and irradiated upon 808 nm laser under stirring. At certain time intervals, 100 μL of the supernatant was taken out through centrifugation, and then used to determine the NO concentration by DAN method.

Intracellular NO detection: Here, we used a commercial intracellular NO fluorescent probe (DAF-FM) to show the intracellular NO generation. In typical, BEL-7402 cells were seeded in glass-bottom dishes at the density of 50 000 cells per dish. After the cells were adherent, Bi_2S_3 or BNN-Bi_2S_3 were added to replace the original medium, and then incubated for 6 h. Cells were treated with DAF-FM solution (5 μM) for 20 min and washed with PBS for 3 times. After loading the fluorescent probe, the cells were irradiated upon 808 nm laser for 10 min. The nuclei were stained by Hoechst 33342. Finally, the stained cells were observed by a confocal microscope (Nikon A1, 40x objective).

Multicellular tumor spheroid model: Firstly, agarose-coated 96-well plates should be prepared. Add 0.09 g of agarose to 6 mL of DMEM in a beaker, and then seal with a lid and autoclave at 115 ^{o}C for 30 min. Before the agarose solution cooling down, add 60 μL agarose

to each well of a 96-well microplate. The agarose would solidify within 30 min. Next, single-cell suspensions would be prepared by mild trypsinization. Add 0.3 mL of Matrigel (Invitrogen) to 12 mL of single-cell suspension (10 000 cells/mL), and then seed 200 μL to each well of agarose-coated 96-well plate. After centrifuge at 1200 rpm for 10 min, the plate was set in humidified atmosphere with 5 % CO_2 in air at 37 ^oC for 7 days.

Detection of NO in MCTS model: Briefly, BEL-7402 cells were cultured for 7 days to form spheroids following the above methods. The medium was replaced by fresh DMEM with BNN-Bi_2S_3 nanoparticles for 24 h. Then, MCTS were stained with both DAF-FM solution (5 μM) and Hoechst 33342 (5 μg mL^{-1}) for 30 min and washed with PBS for 3 times. After irradiation upon 808 nm laser for 10 min, MCTS were transferred to the glass bottom dish carefully, and finally measured by a confocal microscope (Nikon A1, 10x objective). Besides, the fluorescence of NO probe was also detected using flow cytometry. After different treatment, MCTS was trypsinized mildly using 0.1 % trypsin-EDTA, and the single-cell suspension was analyzed using flow cytometry (Accuri c6, BD, USA).

Synergistic effect of PTT and NO in vitro: In vitro cytotoxicity was measured by performing CCK-8 assay, Calcein-AM/PI staining and Annexin V-FITC/PI apoptosis assay. In the typical CCK-8 assay, cells were seeded in 96-well plates at a density of 5 000 cells per well. After cells were adherent, different concentration of Bi_2S_3 or BNN-Bi_2S_3 were added for 6-hour incubation and then irradiated upon 808 nm laser for 10 min. 12 h later, CCK-8 were added and the cells were incubated for another 2 h. The absorbance of the supernatant in each well were read by microplate reader (Multiskan MK3, Thermo) at 450 nm. At least three independent experiments were performed, and each group of different concentration was performed in six replicates. The Calcein-AM/PI staining and Annexin V-FITC/PI apoptosis staining procedures were almost the same as CCK-8 assay. After different treatment, the cells were trypsinized and single-cell suspension was prepared for Calcein-AM/PI staining or Annexin V-FITC/PI apoptosis assay according to the manufacturer's protocol. The results of

Calcein-AM/PI staining were observed through an inverted fluorescence microscopy (IX73, Olympus, Japan). And the results of Annexin V-FITC/PI apoptosis assay were obtained from flow cytometry (Accuri c6, BD, USA).

Autophagy detection: The autophagy level of BEL-7402 cells were detected by Cyto-ID Autophagy Detection Kit (Enzo Life Science) and acridine orange (AO) staining. Typically, BEL-7402 cells were seeded in Cover-glass Bottom Dish at a density of 50 000 cells per dish. After adherence, 40 μg mL^{-1} of Bi_2S_3 or BNN-Bi_2S_3 were added for 6-hour incubation and then irradiated upon 808 nm laser for 15 min. Three hours later, 1 mL fresh medium containing 1 μL autophagy probe was added to replace the original medium, and incubated for 30 min at 37 ^oC. The nuclei were stained with Hoechst 33342, and then the cells were imaged by Nikon A1 confocal laser scanning microscope (CLSM). The AO staining procedure was nearly the same as Cyto-ID Autophagy Detection Assay. After different treatment, the cells were stained with AO (1 μM) for 15 min. CLSM and flow cytometry were both used to analyze the cells.

Immunofluorescent staining: The cytochrome c was detected using immunofluorescent staining method. After different treatment, the cells were fixed with 4 % paraformaldehyde overnight, and blocked with TBST with 5 % BSA for 2 h. Next, the cells were incubated with primary antibody (1:500, Beyotime Biotechnology) at 4 ^oC overnight, and then incubated with FITC-labeled secondary antibody (1:500, Beyotime Biotechnology) at room temperature for 2 h. After washing with TBST, the nuclei were stained with Hoechst 33342, and the cells were observed using CLSM.

Caspase-3 activity assay: The activity of Caspase-3 was measured by Caspase-3 Activity Assay Kit (Beyotime Biotechnology). After treatment, the cells were lysed and the protein was harvested. The whole protein in cell lysates were quantified using Bradford Protein Assay Kit. And the Caspase-3 activity was determined according to the manufacturer's protocol

(Beyotime Biotechnology). The final results were read using microplate reader (Multiskan MK3, Thermo) at 405 nm.

Western blot: BEL-7402 cells were seeded in 6-well plates with the density of 50 000 per well. After 12h, fresh medium containing of Bi_2S_3 or BNN-Bi_2S_3 (40 μg mL^{-1}) was added for 6-hour incubation, and then the cells were irradiated upon 808 nm laser for 15 min. The cells were lysed and the protein was harvested. The cell lysates were separated by SDS-PAGE and transferred onto PVDF membranes. The PVDF membranes were blocked in 5 % BSA solution at 37 ^{o}C for 1 hour, and then incubated with relevant primary antibodies (1:1000 diluted, Cell Signaling Technology) of p62, LC3 and β-actin at 4 ^{o}C overnight. After washing for 3 times, the membranes were incubated with HRP-labeled secondary antibodies (1:2000 diluted, Cell Signaling Technology) at room temperature for 2 h. Finally, the membranes were stained with ECL detection kit (GE Healthcare, Life Sciences) and imaged using FluorChem HD2 Chemi-luminescent Imaging System (ProteinSimple, USA).

In vivo anti-tumor experiment: BEL-7402 tumor bearing female BALB/c nude mice were obtained from Professor Zheng Wang in Cancer Hospital Chinese Academy of Medical Sciences. Mice padding and their food were purchased from Beijing Vitalriver Experimental Animal Technology Co. Ltd. All mice were acclimated to new environment for one week before treatment. When the tumor volume reached nearly 150 mm^3, the mice were divided into 6 groups randomly (PBS, NIR, Bi_2S_3, BNN-Bi_2S_3, Bi_2S_3+NIR, BNN-Bi_2S_3+NIR). The intratumor injection dose of these nanoparticles were 20 mg kg^{-1}, and the NIR exposed time was 10 min (0.35 W cm^{-2}). The temperature of the exposed area was recorded using an infrared thermal imager. The tumor size and mouse weight were recorded every two days. The tumor sizes were measured by a caliper and calculated as volume = length x $(width)^2/2$. Finally, the mice were sacrificed, and tumors and major tissues were dissected to evaluate the therapeutic efficacy and bio-safety by H&E staining, Tunel staining and LC3

immunofluorescence assay. All procedures used were compliant with the Chinese Association for Laboratory Animal Sciences.

Detection of NO in tumor tissue: After different treatment, the tumor bearing mice were sacrificed and the subcutaneous tumors were collected. Then the tumors were lysed using the NO-specific lysis buffer (Beyotime Biotechnology) and tissue grinder. After that, the tissue lysates were collected to detect NO using the Nitric Oxide Assay Kit (Beyotime Biotechnology), according to the manufacturer's instructions. Briefly, 50 μL of each sample was added in the 96-well plate, and then, 50 μL of Griess Reagent I and 50 μL of Griess Geagent II were added into all the wells. After shaking for 5 min, the level of NO was measured by the absorbance at 540 nm, and was calculated for each sample according to the standard curve.

Figures and Tables

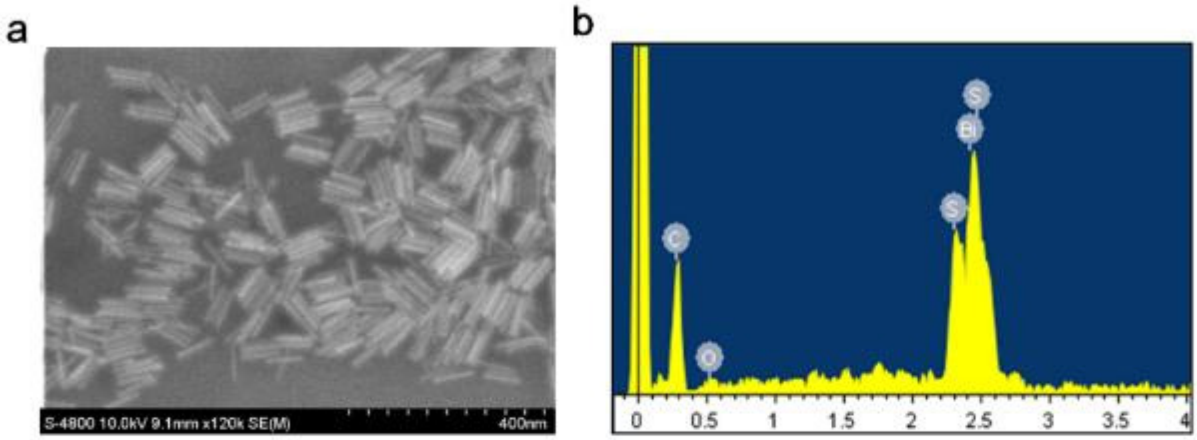

Figure S1. a) SEM image of Bi_2S_3 nanorods. b) EDX spectrum of Bi_2S_3 nanorods.

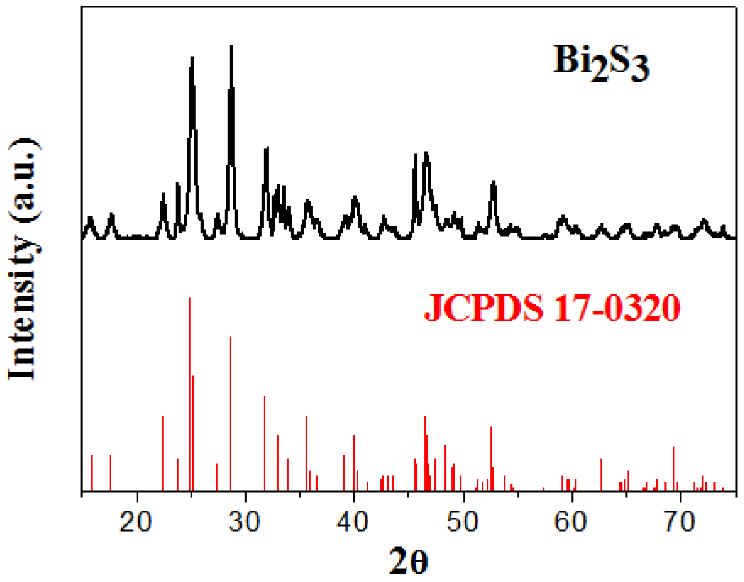

Figure S2. XRD patterns of the as-prepared Bi_2S_3 nanorods. Bottom: the standard pattern for Bi_2S_3 crystals (JPCDS No. 17-0320).

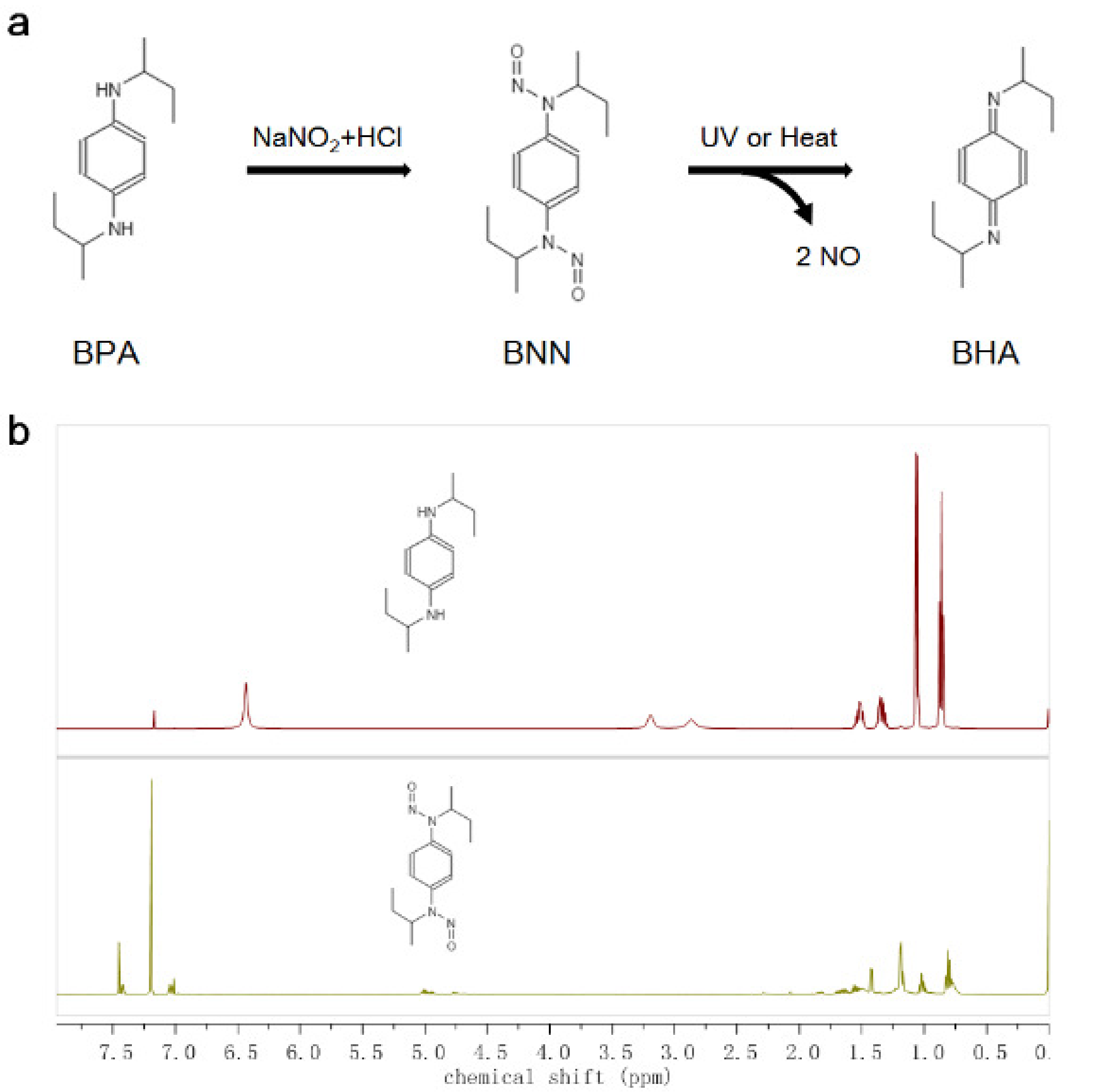

Figure S3. a) The synthetic route of BNN and the decomposition reaction. b) ^{1}H NMR spectra of the reactant BPA and the product BNN: ^{1}H NMR (500 MHz, $CDCl_3$) δ 7.45, 4.78-4.67, 1.82, 1.70-1.6, 1.59-1.49, 1.42, 1.02.

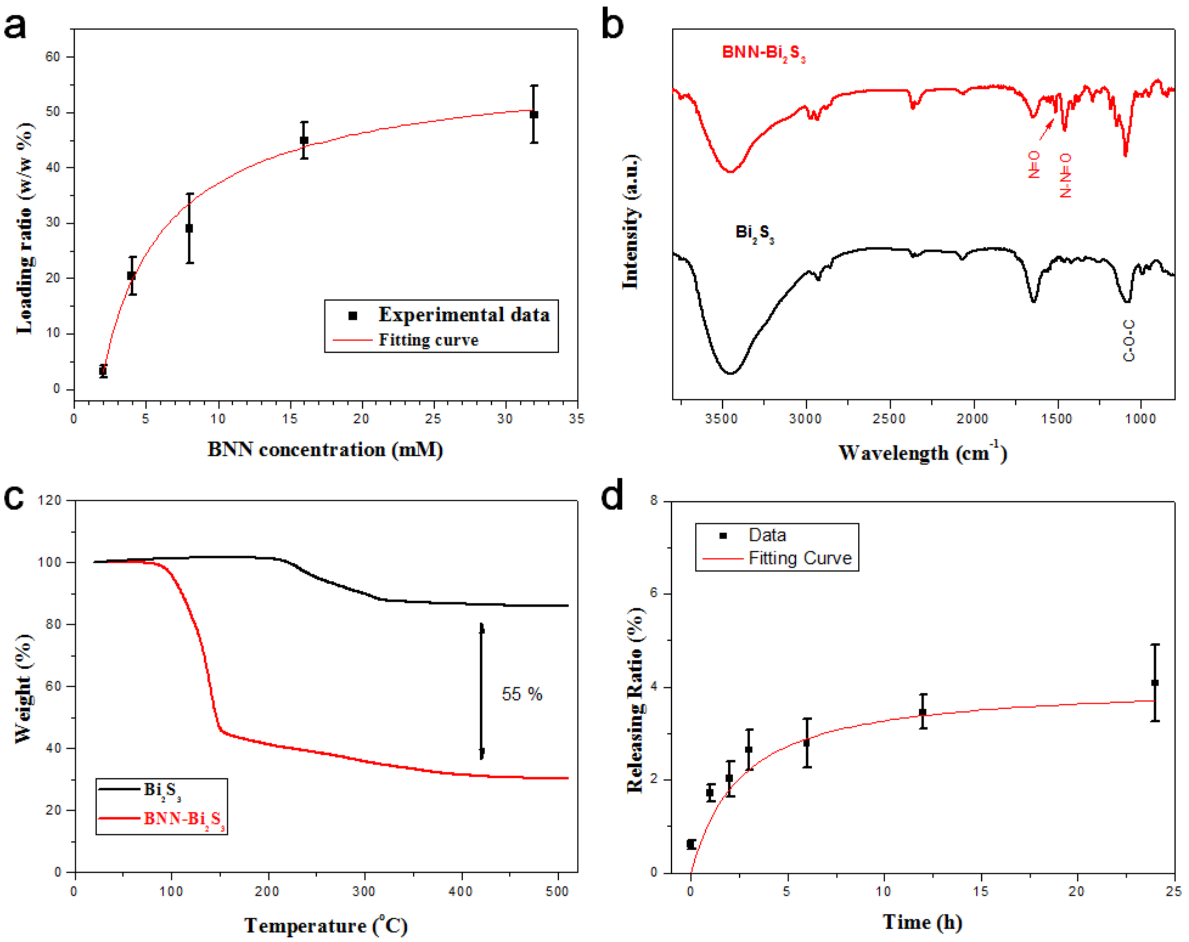

Figure S4. a) Plot of drug loading amount at different concentration of BNN. b) FT-IR spectra of Tween-20 coated Bi_2S_3 (black) and BNN-Bi_2S_3 (red). c) Thermogravimetric (TG) curves of Bi_2S_3 (black) and BNN-Bi_2S_3 (red). d) BNN releasing curve from BNN-Bi_2S_3 at 37 °C.

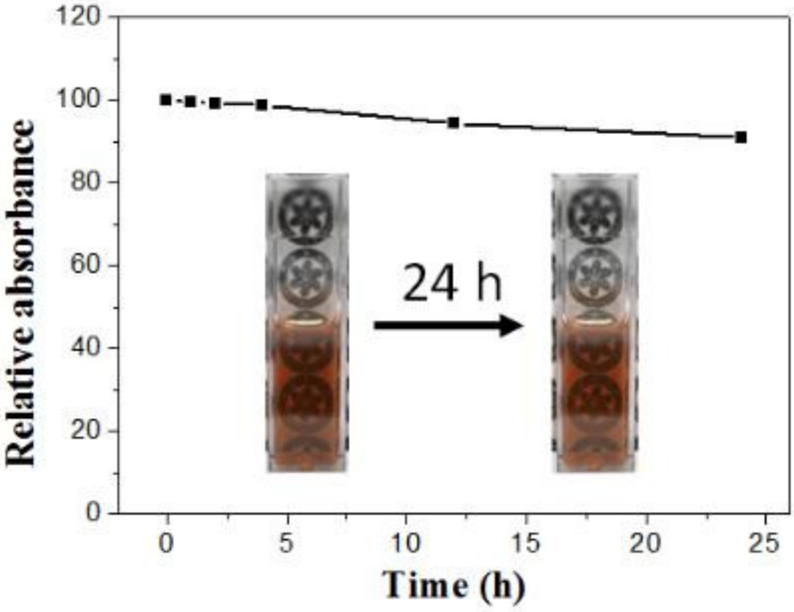

Figure S5. The relative absorbance of the supernatant of BNN-Bi_2S_3 dispersed in PBS solution during 24 h standing. Inset: digital photos of BNN-Bi_2S_3 dispersed in PBS solution.

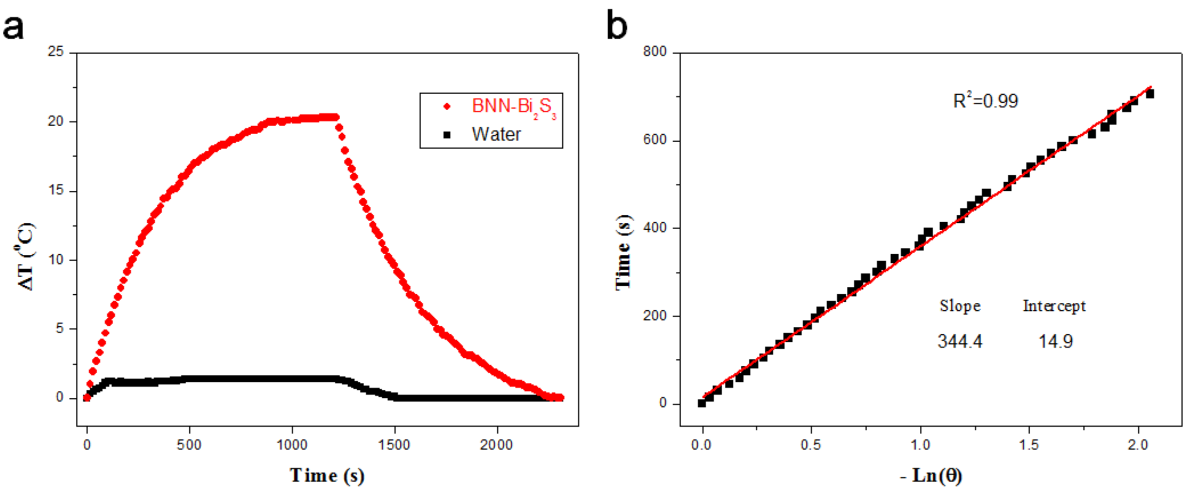

Figure S6. a) Photothermal effect of the as-prepared BNN-Bi_2S_3 dispersion and DI water under 808 nm irradiation. The laser was shut off after irradiation for 20 min. b) Plot of cooling period (after 800 s) versus negative natural logarithm of driving force temperature. Time constant (τs) for heat transfer from the system is determined to be 344.4 s. The photo-thermal conversion efficiency of BNN-Bi_2S_3 is about 33.7 %.

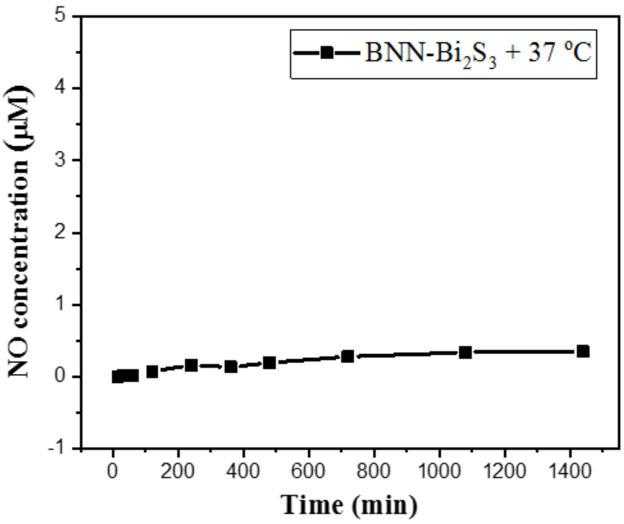

Figure S7. The amount of released NO from BNN-Bi_2S_3 in PBS at 37 ^oC.

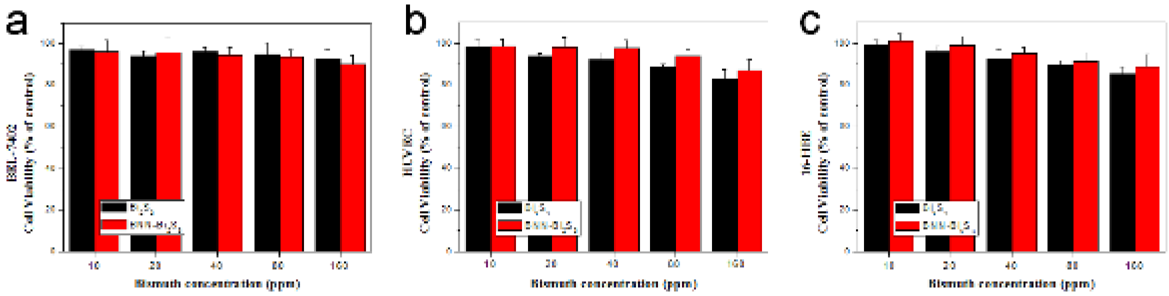

Figure S8. Cytotoxicity evaluation of Bi_2S_3 and BNN-Bi_2S_3 in a) human hepatocellular carcinoma cell line BEL-7402, b) human primary umbilical vein endothelial cell line HUEVC and c) human bronchial epithelial cell line 16-HBE.

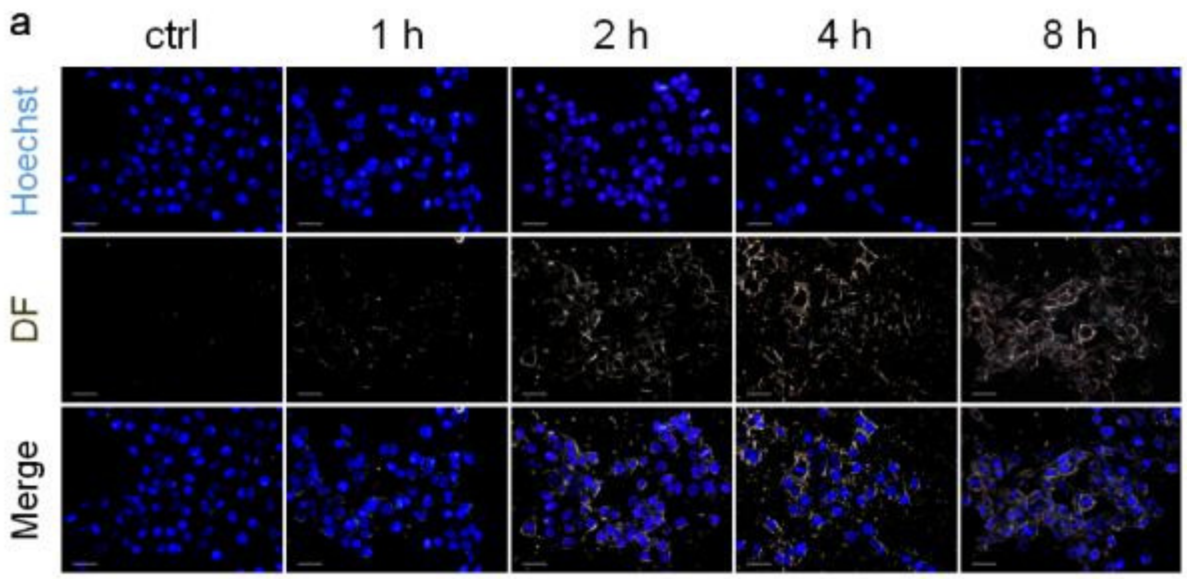

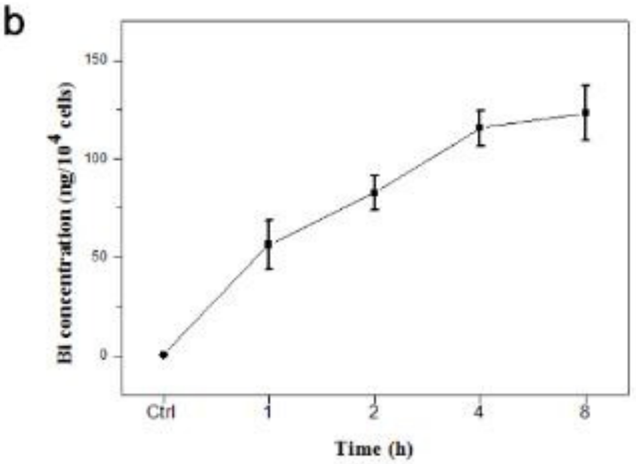

Figure S9. a) Dark-field optical microscopy images of cells incubated with Bi_2S_3 for different times. Cells were stained with Hoechst 33342. All the scar bars are 50 µm. b) Time-dependent cellular uptake of Bi_2S_3 determined by inductively coupled plasma mass spectrometry (ICP-MS) after incubation. The concentration of Bi_2S_3 is 10 µg mL^{-1}.

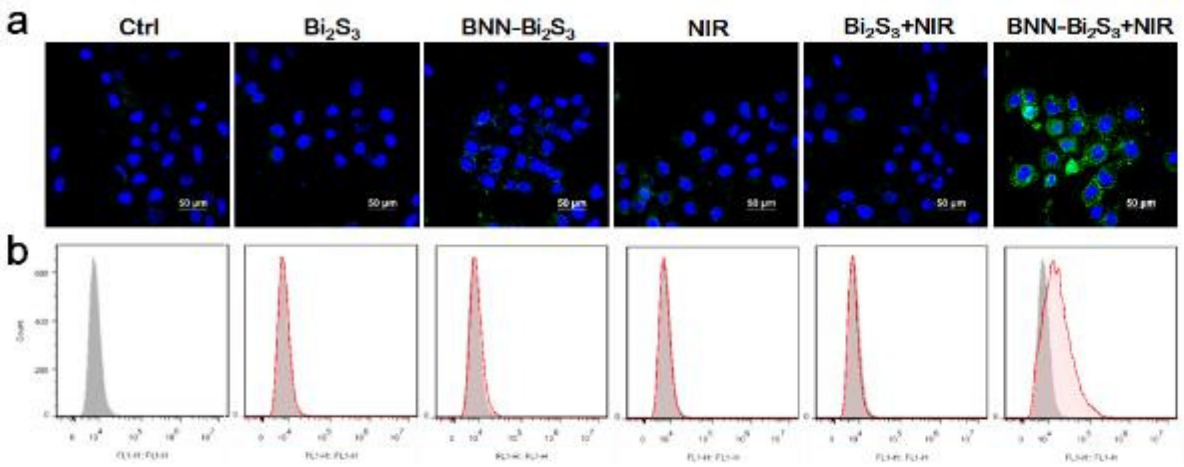

Figure S10. a) Fluorescence images of BEL-7402 cells staining with Hoechst 33342 (blue) and DAF-FM (green) upon being treated with PBS, Bi_2S_3 or BNN-Bi_2S_3 in dark or under 808 nm irradiation (0.5 W cm^{-2}, 10 min). b) Flow cytometry analysis of DAF-FM intensity in BEL-7402 cells with different treatment.

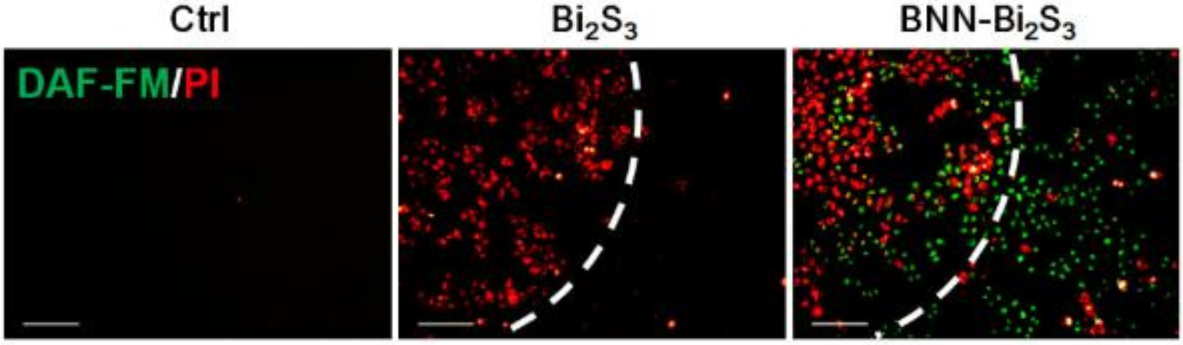

Figure S11. Fluorescent images of BEL-7402 cells with different treatment. The cells were pre-stained with DAF-FM (green) and cultured with PI under NIR irradiation. The white dash circle indicated the laser spot. The scale bars are 100 μm. The concentration of Bi_2S_3 or BNN-Bi_2S_3 nanoparticles is 20 μg mL^{-1}. The irradiation power is 1.0 W cm^{-2}, for 20 min.

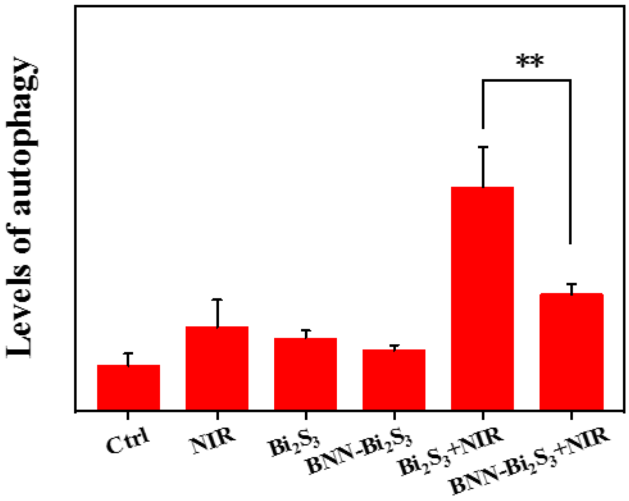

Figure S12. Flow cytometer analysis of CYTO-ID probe intensity in BEL-7402 cells with different treatment. $^{**}P < 0.01$.

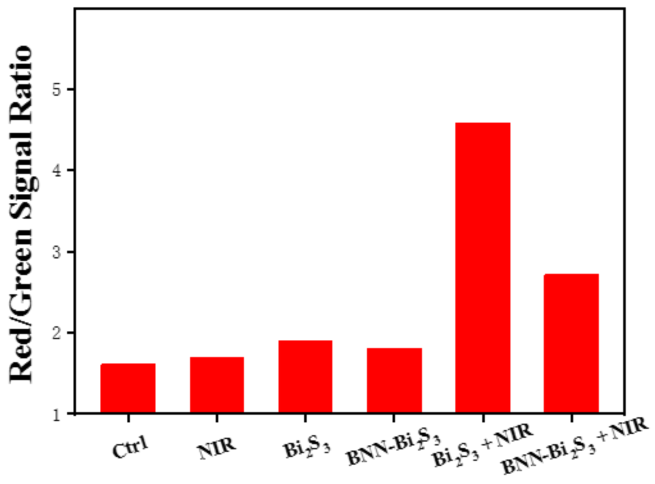

Figure S13. Flow cytometer analysis of AO intensity in BEL-7402 cells with different treatments.

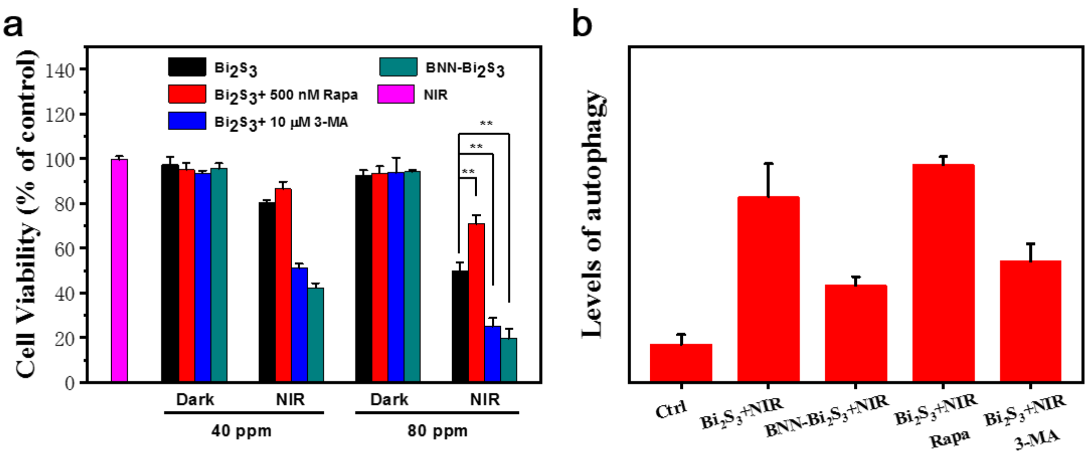

Figure S14. a) Cell viability of BEL-7402 cells with different treatment. The NIR laser power density is 1.0 W cm^{-2} (10 min). b) Autophagy detection of BEL-7402 cells with different treatment. The data points are shown as mean value and standard deviation, n=3. P values were calculated by Student's t test (**$P < 0.01$).

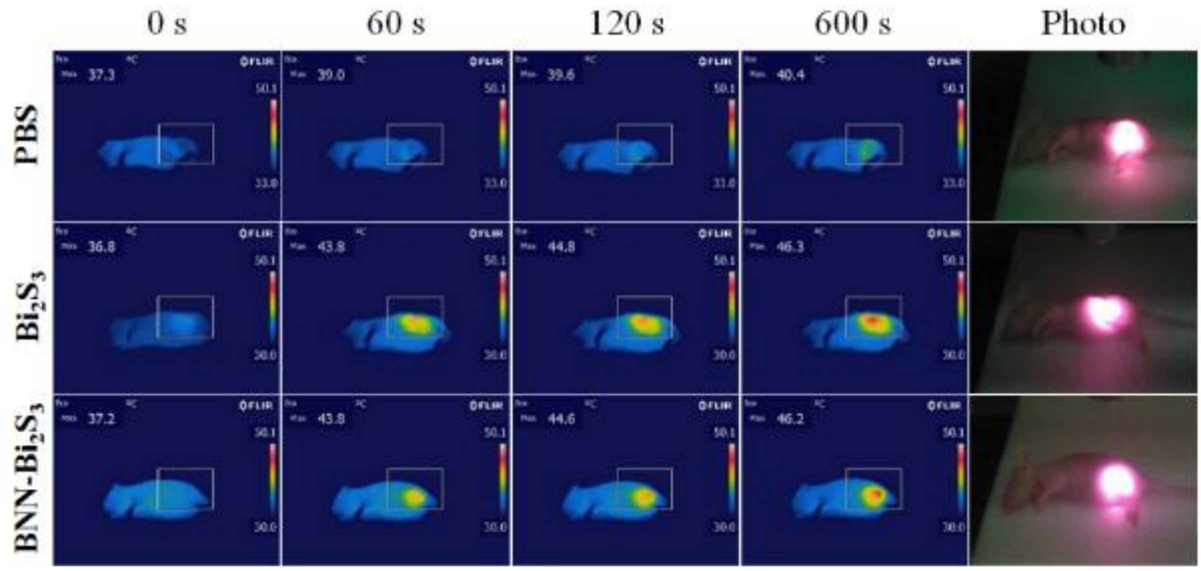

Figure S15. Infrared thermal images of BEL-7402 tumor-bearing mice with injection of PBS, Bi_2S_3 or BNN-Bi_2S_3 under the 808 nm laser irradiation taken at different time intervals. The NIR laser power density is 0.35 W cm^{-2}.

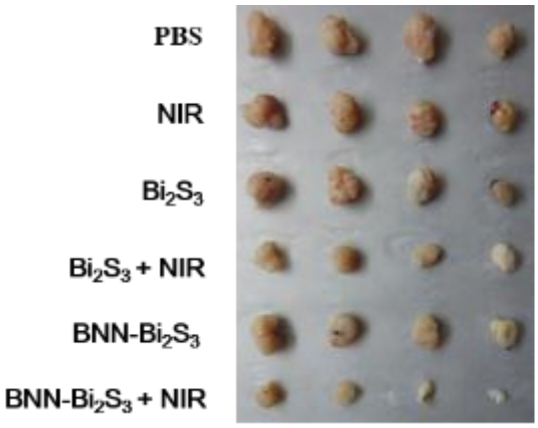

Figure S16. Photo of the whole tumors collected from different groups at the end of treatment.

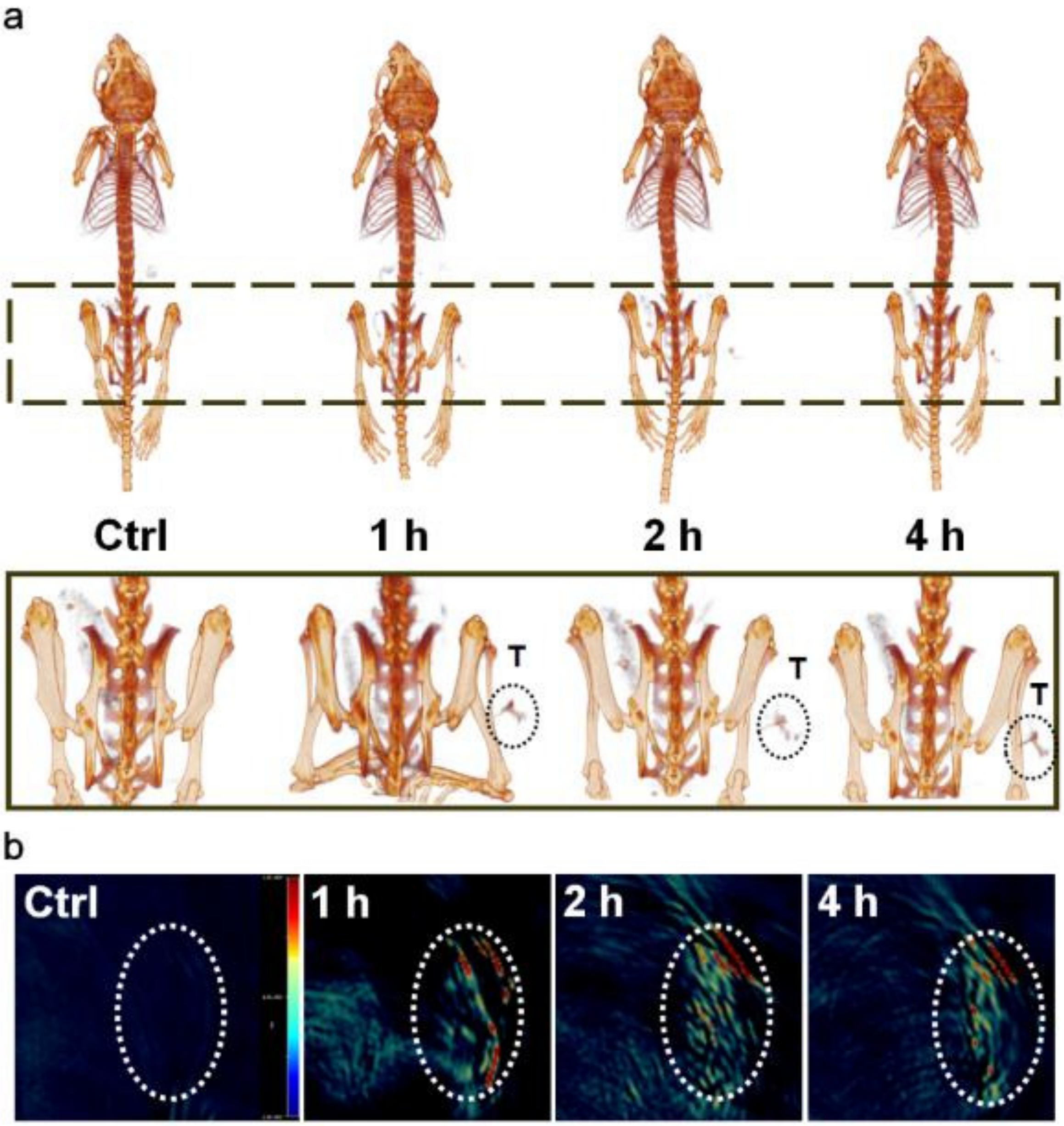

Figure S17. (a) In vivo X-ray CT imaging in BEL-7402 tumor-bearing mice. (b) In vivo MSOT imaging in BEL-7402 tumor-bearing mice.

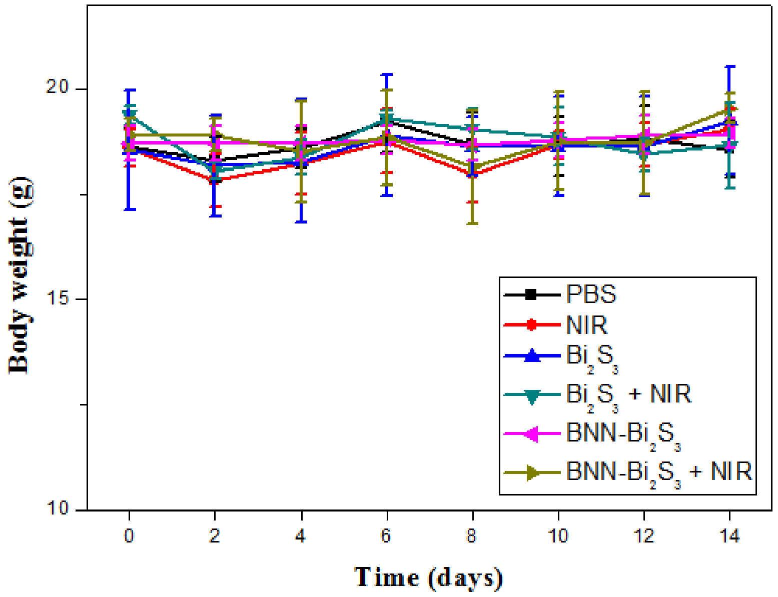

Figure S18. Body weight of BEL-7402 bearing mice as a function of time for different treatment groups.

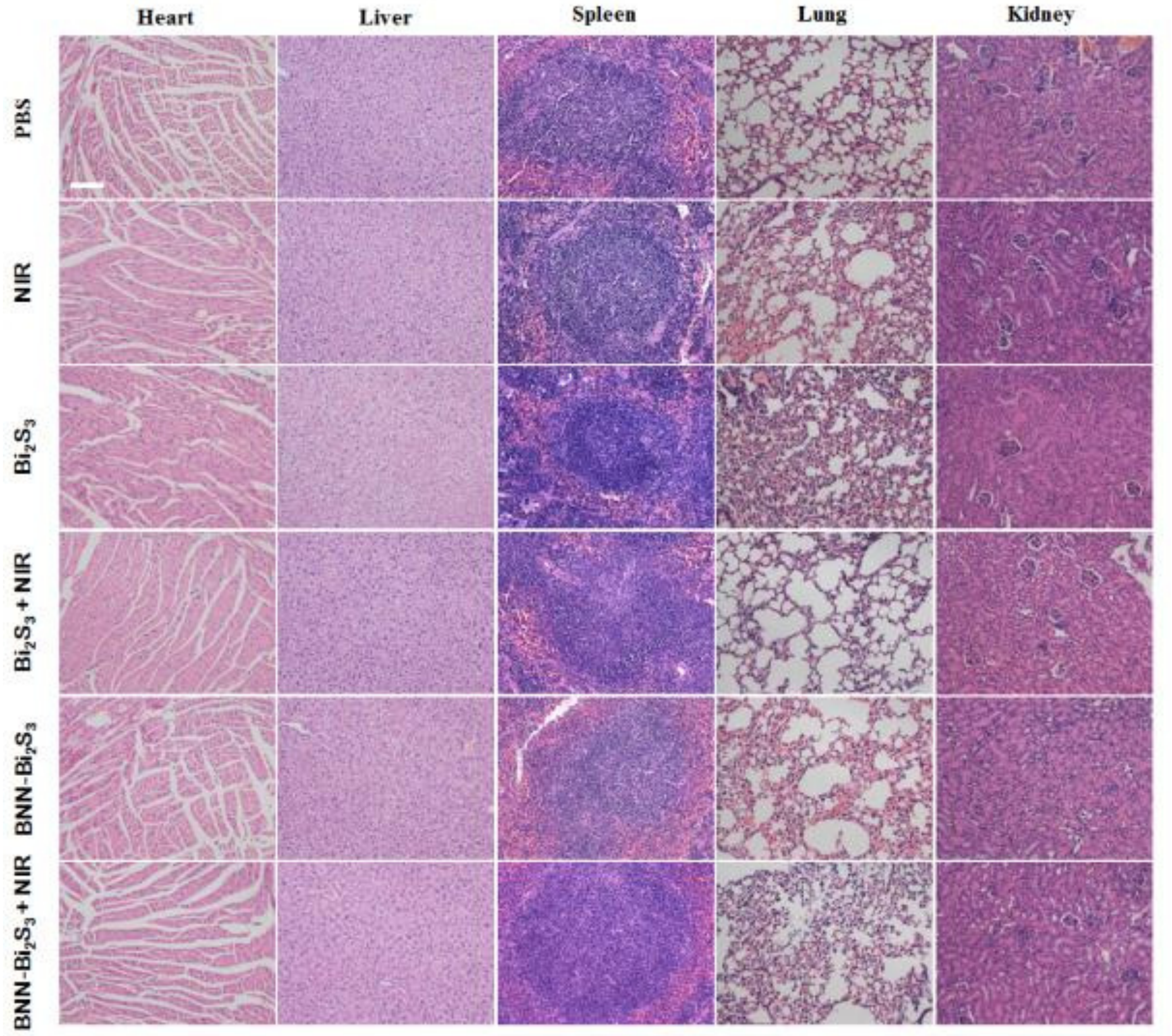

Figure S19. H&E stained tissue sections from the major organs (heart, liver, spleen, lung, and kidney) of mice after different treatment. The scale bar is 50 μm.

Table S1. Side-by-Side Comparison of BNN-Bi_2S_3 with other NIR-triggered NO delivery system based on UCNPs.

**NO delivery system**	**NIR-triggered**	**NO donor loading ratio (wt %)**	**Max concentration (μM under 1 W cm^{-2})**
RBS-UCNPs	Yes	≤ 10%	≤ 1
BNN-UCNPs	Yes	≥ 30%	≤ 2
**BNN-Bi_2S_3**	**Yes**	**≥ 50%**	**10**
